# Supplementary material for: Optical imaging of strain in two-dimensional crystals
Source: Nat Commun. 2018 Feb 6;9:516. doi: 10.1038/s41467-018-02830-y (PMC5802795; doi:10.1038/s41467-018-02830-y)
Supplement: Supplementary file 1 — Supplementary Information [file 41467_2018_2830_MOESM1_ESM.pdf]

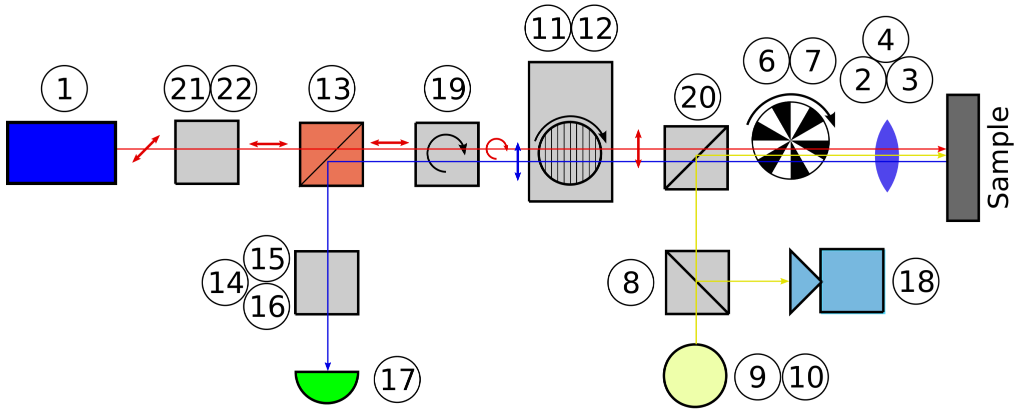

Supplementary Figure 1 | Experimental setup. Schematic illustration of the experimental setup. The red/blue/yellow lines indicate the 800 nm laser beam, 400 nm second harmonic signal and the white light, respectively. 1, Ti:sapphire laser; 2, 100× objective lens; 3, 20× objective lens; 4, objective lens turret; 5, mirror; 6, optical chopper; 7, chopper wheel; 8, beam splitter; 9, fiber collimator; 10, fiber illuminator; 11, motorized rotation stage; 12, linear polarizer; 13, dichroic mirror; 14, lens tube filter holder; 15, short pass filter; 16, band pass filter; 17, amplified photodetector; 18, CMOS camera; 19, quarter wave plate; 20, removable beam splitter; 21, half wave plate; 22, Glan-Laser polarizer.

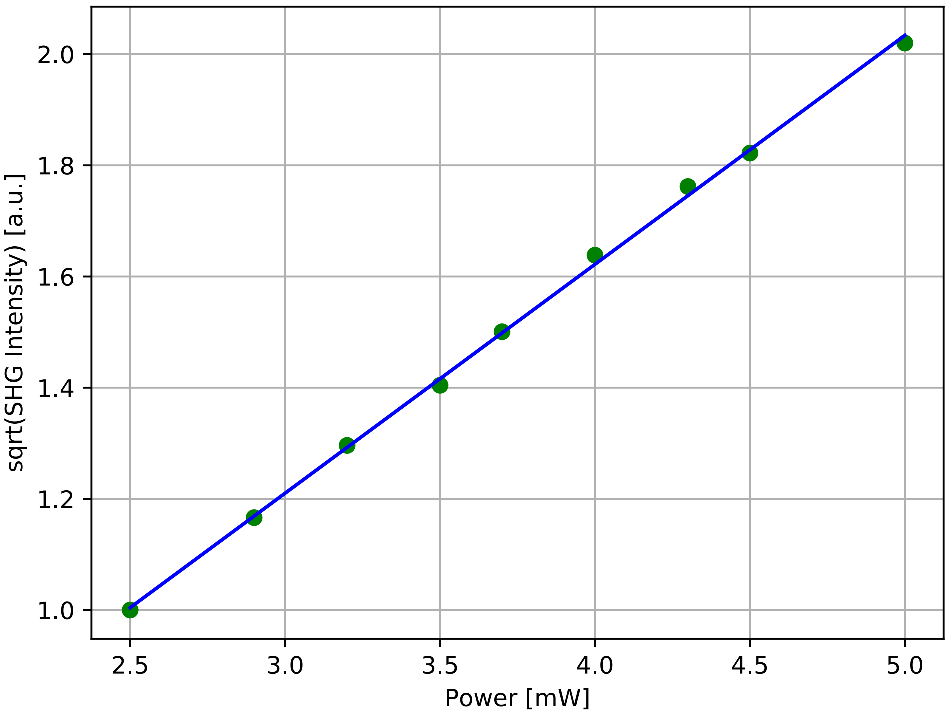

Supplementary Figure 2 | Power dependence. Excitation power dependence of the SHG amplitude in a MoS_2 monolayer (symbols: measurement; line: fit).

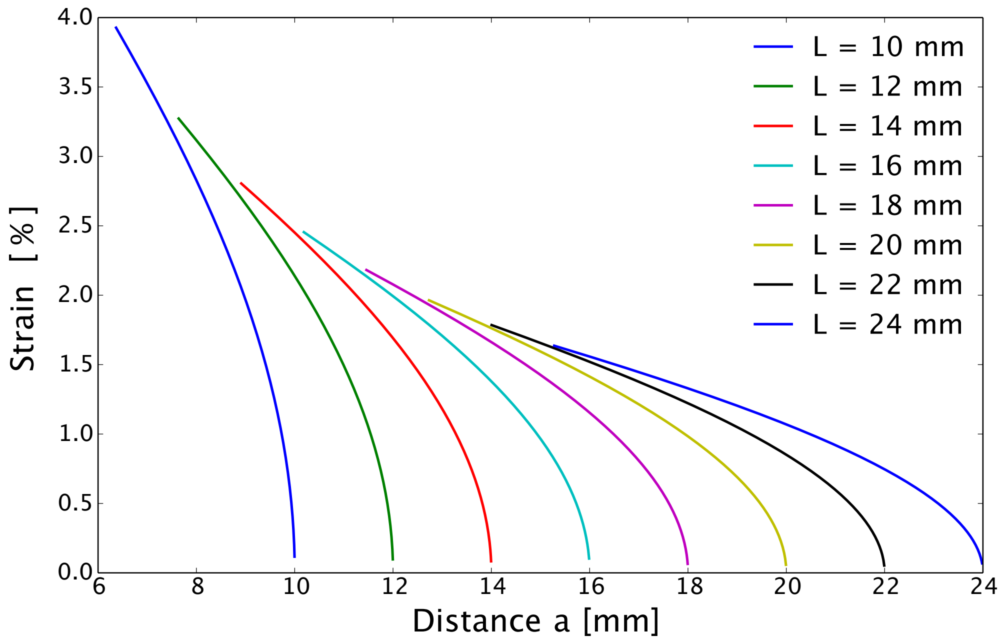

Supplementary Figure 3 | Bending strain. Numerically calculated two-point bending strain for different sample lengths L. Sample thickness $d = 0.25$ mm.
